# Low-intensity and moderate exercise training improves autonomic nervous system activity imbalanced by postnatal early overfeeding in rats

**DOI:** 10.1186/1550-2783-11-25

**Published:** 2014-06-02

**Authors:** Wilson Rinaldi, Rodrigo Mello Gomes, Dionízia Xavier Scomparin, Sabrina Grassiolli, Tatiane Aparecida Ribeiro, Gabriel Sergio Fabricio, Luiz Felipe Barella, Audrei Pavanello, Amanda Bianchi Trombini, Paulo Cezar de Freitas Mathias, Júlio Cezar de Oliveira

**Affiliations:** 1 Department of Physical Education, State University of Maringá, Maringá, PR, Brazil; 2 Department of Physiological Sciences, State University of Maringá, Maringá, PR, Brazil; 3 Department of General Biology, State University of Ponta Grossa, Ponta Grossa, PR, Brazil; 4 Department of Biotechnology, Genetics and Cell Biology, State University of Maringá, Maringá, PR, Brazil

**Keywords:** Moderate exercise training, Overfeeding-induced obesity, Autonomic nervous system, Parasympathetic nervous system, Glucose homeostasis

## Abstract

**Background:**

Postnatal early overfeeding and physical inactivity are serious risk factors for obesity. Physical activity enhances energy expenditure and consumes fat stocks, thereby decreasing body weight (bw). This study aimed to examine whether low-intensity and moderate exercise training in different post-weaning stages of life is capable of modulating the autonomic nervous system (ANS) activity and inhibiting perinatal overfeeding-induced obesity in rats.

**Methods:**

The obesity-promoting regimen was begun two days after birth when the litter size was adjusted to 3 pups (small litter, SL) or to 9 pups (normal litter, NL). The rats were organized into exercised groups as follows: from weaning until 90-day-old, from weaning until 50-day-old, or from 60- until 90-days-old. All experimental procedures were performed just one day after the exercise training protocol.

**Results:**

The SL-no-exercised (SL-N-EXE) group exhibited excess weight and increased fat accumulation. We also observed fasting hyperglycemia and glucose intolerance in these rats. In addition, the SL-N-EXE group exhibited an increase in the vagus nerve firing rate, whereas the firing of the greater splanchnic nerve was not altered. Independent of the timing of exercise and the age of the rats, exercise training was able to significantly blocks obesity onset in the SL rats; even SL animals whose exercise training was stopped at the end of puberty, exhibited resistance to obesity progression. Fasting glycemia was maintained normal in all SL rats that underwent the exercise training, independent of the period. These results demonstrate that moderate exercise, regardless of the time of onset, is capable on improve the vagus nerves imbalanced tonus and blocks the onset of early overfeeding-induced obesity.

**Conclusions:**

Low-intensity and moderate exercise training can promote the maintenance of glucose homeostasis, reduces the large fat pad stores associated to improvement of the ANS activity in adult rats that were obesity-programmed by early overfeeding.

## Background

Obesity has reached epidemic proportions in many of the developed countries of the world. This phenomenon is frequently ascribed to the combination of excess food consumption and decreased physical activity [1]. The habits acquired in childhood have a major impact on adult life, and in most cases, determine the state of health during adulthood, particularly with respect to metabolic and endocrine disturbances. Extensive findings indicate that metabolic programming occurs in humans, and this metabolic programming is correlated with the increased risk of adverse health effects, such as diabetes, hypertension and cardiovascular disease [2-4]. Animal studies demonstrate that nutritional programming during the early periods of postnatal life has numerous long-term growth consequences [5-9]. The intrauterine and lactation phases of life are crucial periods in brain growth and development processes; it is during these stages that critical events of cell migration and differentiation occur [10,11]. Nutritional insults, by either low or overfeeding, on these stages may be responsible for the changes in the hypothalamic pathways involved in metabolic balance and energy homeostasis [12,13]. As reported, early overfeed-programmed obese rats exhibit disrupted neuronal firing in the central nervous regulation of body weight (bw) [14].

Several maternal environmental insult conditions have been linked to obesity in both human and rodent offspring, which, in turn, has been shown to affect neural development. Interestingly, both maternal caloric deprivation and maternal overfeeding can leads to metabolic syndrome in offspring [15,16]. Overfeeding and obesity are often accompanied by alterations in both sympathetic and parasympathetic autonomic function. Several lines of evidence support the hypothesis that derangements in the autonomic nervous system (ANS) play an important role in the development of obesity [17,18]. As reported, other different models of obesity display imbalanced function of the ANS [19,20]. The sympathetic and parasympathetic nervous systems are critical in the coordination of the catabolic and anabolic responses, respectively. In response to physical activity, glucose uptake is increased in the adipose and skeletal muscle cells; which happens regardless of insulin action [21,22].

The major metabolic changes induced by exercise training are caused by the enhancement of sympathetic tonus. Adrenodemedullated rats that were submitted to swimming training showed low fat mobilization; where was showed that the long-term exercise training led to the mobilization of fat, and the fat gains in these adrenodemedullated rats were more consistent [23]. Thus, it is important to keep in mind that the exercise training may increase the basal metabolism to promote further increases in fat store consumption, even at rest. As previously reported by our group, the low-intensity and moderate swimming training was able to attenuate obesity onset induced by monosodium L-glutamate (MSG) in mice. However, the benefits of this protocol were observed only in cases where exercise was started early, soon after weaning [24].

Rat’s litter size reduction provokes overfeeding behavior in suckling pups, which induces a high chow intake post-weaning and subsequent obesity. The early overfeeding model of obesity is interesting because the development of obesity in childhood and adolescence is highly correlated with the onset of the metabolic syndrome in adulthood [25,26]. In the present study, we aimed to evaluate the effects of low-intensity and moderate exercise training applied during different post-weaning development stages on modulating the ANS activity and its role on blocks the obesity progression in rats that were early overfeed.

## Materials

### Ethical approval

All experiments were undertaken according to the norms established by the Brazilian Association for Animal Experimentation (COBEA) and were previously approved by the Ethics Committee in Animal Research of the State University of Maringá (protocol number 084/2009).

### Animals and obesity induction

Sets of 3 female and 1 male Wistar rats, 70 days old, were mated. After 1 week, the pregnant rats were separated. On the 2^nd^ day of life, the size of the normal litters (NL) was set to 9 pups; while the small litter (SL) size was set to 3 pups. After weaning (21^st^ day), males were selected, and all females were discharged. Young male rats from the NL and SL groups were randomly chosen for exercise. Animals received water and a commercial diet (Nuvital®; Curitiba/PR, Brazil) *ad libitum*. During all protocol stages, the animals were placed in an environmentally controlled room (23 ± 3°C; 12 hour light/dark photocycle (07:00–19:00 h).

### Exercise training protocol

Rats from the NL exercised (NL-EXE) and SL exercised (SL-EXE) groups were trained on an animal treadmill (model ET-2000 Insight®; Ribeirão Preto/SP, Brazil). Three trained groups were established: exercise beginning after weaning in 21-day-old rats and ending at 90-day-old (EXE_21–90_); exercise beginning at weaning and stopped at 50-day-old (EXE_21–50_); and exercise beginning at 60 days old and ending at 90 days old (EXE60_60–90_). Another group of NL and SL rats did not exercise at all (N-EXE). Running protocols, including running speeds and times, were set to induce moderate-intensity exercise training, promoting a 50-70% total oxygen uptake (VO_2max_) for each animal, independent of age. The running protocols used have been described previously [27,28] with some modifications. The anaerobic threshold of the rats is approximately 20 m.min^-1^ and was used to delimit the maximal velocity reached in the training program. This protocol was intended to guarantee the same aerobic exercise intensity across all ages as the animals grew.

### Adaptation period of exercise protocol

Exercise sessions lasted 10 min on the first day of the adaptation period, and the rats were run at a velocity of 10 m × min^-1^. The sessions were increased to 20 min at 12 m × min^-1^ for the subsequent sessions. The rats in the group exercised from days 21–90 had an adaptation time of two weeks, and the rats in the 21–50 day group and the 60–90 day group had an adaption time of one week, as previously reported [27]. The running sessions were performed in the afternoon. The rats that did not adapt were eliminated.

### Training period

**Figure 1:**
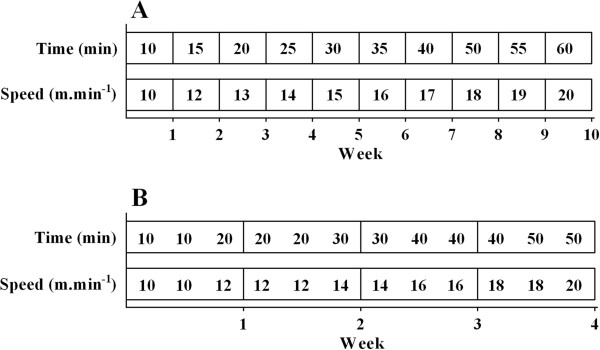
**Schematical figure depicting the treadmill exercise training protocol.** The time sessions, speed and duration depict the intensity of exercise training throughout the period in which exercise training protocol was performed. Exercise training protocol applied from 21- until 90-days-old **(A)**; and applied from 21- until 50-days-old or from 60- until 90-days-old **(B)**.

In the EXE_21–90_ groups, the initial training speed was 12 m × min^-1^ for 20 min and was increased to 20 m × min^-1^ for 60 min over ten weeks (Figure 1A). The initial speed of the EXE_21–50_ and EXE_60–90_ groups was 12 m × min^-1^ for 20 min and was increased to 20 m × min^-1^ for 50 min over four weeks (Figure 1B). The variation of the training period time and velocity was adjusted for each protocol and their specific sessions.

### Food intake

After weaning, rats from all groups were weighed, and food intake was determined every week by non-ingested chow. Food intake was calculated for each animal as chow consumed divided by bw. The total area under the curve (AUC) of food consumption throughout experimental protocol was calculated.

### Intravenous glucose tolerance test (ivGTT)

At 91-day-old, rats from all groups underwent a surgery for the silicone cannula implantation into the right jugular vein, as previously described [29]. At 24 h after the surgery, and after to be fasted overnight (12 h; 7:00 PM to 7:00 AM) the rats received a glucose infusion (1 g/kg bw) by a cannula implanted in the right jugular vein. Blood samples were collected in heparinized syringes at 0 (before glucose administration), 5, 15, 30 and 45 min after the glucose administration. Plasma samples were stored at -20°C for determination of glucose concentrations by the glucose oxidase method (Gold Aanlisa®; Belo Horizonte/MG, Brazil). The AUC of glycemia throughout the ivGTT was calculated.

### Autonomic nerves activity assessment

At 91-day-old, a batch of rats from all of the experimental groups, after to be fasted overnight was subsequently anesthetized with thiopental (45 mg/kg bw). As previously described [29], surgical longitudinal incisions were made on the anterior cervical region. Under the dissection microscope, the nerve bundle of the left superior branch of the upper vagus nerve was severed from the carotid artery close to the trachea. The nerve trunk was pulled with a fine cotton line, and a pair of recording silver electrodes (0.6 mm diameter), similar to a hook, were placed under the nerve. The nerve was covered with silicone oil to prevent dehydration. The electrode was connected to an electronic device (Bio-Amplificator, Insight®; Riberão Preto/SP, Brazil), which amplified the electrical signals up to 10,000 times, and the low and high frequencies, 1–80 kHz, were filtered. The neural signal output was acquired by an Insight interface (Insight®; Riberão Preto/SP, Brazil), viewed online and stored by a personal computer running software developed by Insight (Bio-Amplificator, Insight®; Riberão Preto/SP, Brazil). During all data acquisition, the animals were placed in a Faraday cage to avoid any electromagnetic interference. Nerve activity was analyzed as the number of spikes during 5 sec. After stabilization of the signal for 2 min, 20 record frames of 15 sec from each animal were randomly chosen for spike counting. The average number of spikes was used as the nerve firing rate for each rat.

The branch of the sympathetic nerve from the lumbar plexus that innervates the retroperitoneal white fat tissue, which may be called the greater splanchnic nerve, was dissected from another batch of anesthetized rats from all experimental groups, as described above. The electrode was placed under the greater splanchnic nerve, close to the retroperitoneal area. Firing rates from the nerve were obtained as described for the vagus nerve.

### Obesity assessment

After all experimental procedure, as described above, both exercised and no-exercised rats were anaesthetized by an intraperitoneal injection of pentobarbital sodium (thiopental 45 mg/kg bw) and killed by cervical dislocation. The retroperitoneal fat pads were removed and weighed. The fat mass of this tissue was used as a simple reliable estimation of total body fat in normal and obese rodents.

### Statistical analysis

The results are expressed as the mean ± SEM. Data were submitted to variance analysis (one-way ANOVA). In the case of analyses with a significant F, the differences between the means were evaluated by Tukey’s test. Probability values less than .05 (p < .05) were considered statistically significant. Tests were performed using GraphPad Prism version 5.0 for Windows (GraphPad Software Inc., San Diego/CA, USA).

## Results

### Biometric parameters

As shown in Table 1, the SL-N-EXE group exhibited larger bw (10%) when compared to the NL-N-EXE group (p < .01). In the NL-EXE_21–90_ group, exercise reduced the bw by 13% compared to the NL-N-EXE group (p < .05). No differences were observed among the NL-N-EXE, NL-EXE_21–50_ and NL-EXE_60–90_ groups. In contrast, the SL-EXE_21–90_, SL-EXE_21–50_ and SL-EXE_60–90_ groups exhibited bw reductions around of 10%, in relation to the SL-N-EXE (p < .05).

**Table 1:** Effect of low-intensity and moderate exercise training during different ages on fasting glycemia and biometric parameters

		**Body weight (g)**	**AUC food intake (g/100 g of bw)**	**Retroperitoneal fat pad (g/100 g bw)**	**Glycemia (mg/dL)**
**N-EXE**	**NL**	386.7 ± 4.2	179.0 ± 5.1	0.88 ± 0.02	81.8 ± 3.0
	**SL**	423.1 ± 6.4**	205.0 ± 4.2**	1.66 ± 0.03**	109.4 ± 2.2**
**EXE**_**21–90**_	**NL**	334.5 ± 4.4*	180.5 ± 3.2	0.66 ± 0.02*	83.4 ± 2.1
	**SL**	384.6 ± 5.0^#^	204.8 ± 1.3	1.07 ± 0.02^#^	89.5 ± 2.9^#^
**EXE**_**21–50**_	**NL**	395.8 ± 4.9	193.3 ± 3.2	0.76 ± 0.04	78.2 ± 1.9
	**SL**	385.3 ± 10.1^#^	206.5 ± 1.5	1.21 ± 0.04^#^	94.2 ± 3.4^#^
**EXE**_**60–90**_	**NL**	387.7 ± 3.9	185.0 ± 5.7	0.73 ± 0.04	86.2 ± 3.2
	**SL**	380.2 ± 9.6^#^	209.8 ± 4.7	0.97 ± 0.02^#^	87.2 ± 1.5^#^

All values are expressed as the mean ± SEM of 10–16 rats from each experimental group. *p < .05 and **p < .01 v.s*.* NL-N-EXE; ^#^p < .05 v.s*.* SL-N-EXE; by one-way ANOVA followed by the Tukey’s test.

As showed in Table 1, the retroperitoneal fat pad content was larger in the SL-N-EXE group (88%) compared to the NL-N-EXE group (p < .01). Moderate exercise training reduced the retroperitoneal fat pad in the NL-EXE_21–90_ group by 25% (p < .05), whereas no differences were observed among the NL-N-EXE, NL-EXE_21–50_ and NL-EXE_60–90_ groups. In all of the SL-EXE groups (21–90, 21–50 and 60–90), moderate exercise training reduced the weight of the retroperitoneal fat pads (35%, 27% and 41%, respectively) in relation to those of the SL-N-EXE group (p < .05).

### Food intake

The AUC of food intake exhibited significant differences between the NL-N-EXE and the SL-N-EXE groups (p < .05; Table 1). Exercise training did not change food intake in either group (NL-EXE and SL-EXE), independent of the period in which exercise protocol was applied (21–90, 21–50 or 60–90).

### Glycemic homeostasis

When compared with the NL-N-EXE group, the fasting blood glucose levels were reduced by 34% in the SL-N-EXE group (p < .05; Table 1). Exercise altered fasting plasma glucose concentrations independent of the period in which protocol was applied, decreasing levels by 18%, 14% and 20% in the SL-EXE_21–90_, SL-EXE_21–50_ and SL-EXE_60–90_ groups, respectively, when compared to the SL-N-EXE group (p < .05; Table 1). Exercise did not change fasting blood glucose levels in the NL-EXE groups compared to NL-N-EXE group (Table 1).

**Figure 2:**
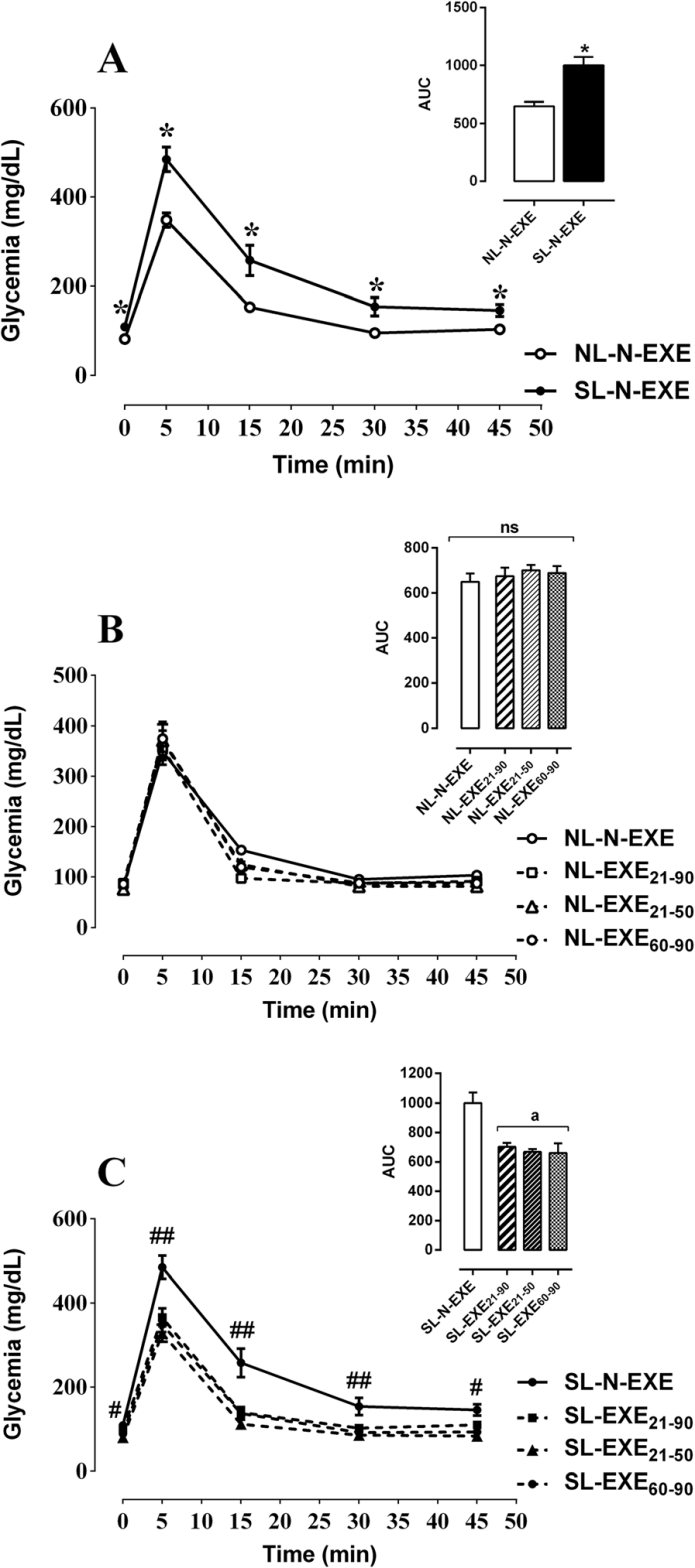
**Intravenous glucose tolerance test (ivGTT).** All values are expressed as the mean ± SEM of 12–15 rats for each experimental group. **(A)** NL-N-EXE versus SL-N-EXE; **(B)** NL-N-EXE versus all NL-EXE groups and **(C)** SL-N-EXE versus all SL-EXE groups. Symbols on the lines as well as letters on the bars represents the statistical difference by one-way ANOVA followed by Tukey’s test among groups. *p < .01 for NL-N-EXE v.s*.* SL-N-EXE, (Figure 2**A**); ^##^p < .01, ^#^p < .05 for each one of SL-EXE group v.s*.* SL-N-EXE, (Figure 2**C**). The upper panel of each figure represents the area under the curve of glycemia during the ivGTT. (ns) Represents no statistical difference in the Figure 2**B** and **(A)** represents SL-N-EXE group in the Figure 2**C**.

Throughout the ivGTT, the SL-N-EXE group exhibited plasma glucose levels higher than those of the NL-N-EXE group (Figure 2A). As shown by the AUC (inset of the Figure 2A), postnatal early overfeeding in rats increased glycemia by 54% during the ivGTT when compared to the NL-N-EXE group (p < .05). No significant difference was observed between the NL-N-EXE and NL-EXE groups (Figure 2B). However, the exercise training was able on improves the glucose intolerance of the SL rats. As showed in the inset of the Figure 2C, the SL-EXE (SL-EXE_21–90_, SL-EXE_21–50_ and SL-EXE_60–90_) groups exhibited lower plasma glucose levels in relation to the NL-N-EXE group, which were similar to those of the NL-N-EXE rats.

### Autonomic nervous activity

**Figure 3:**
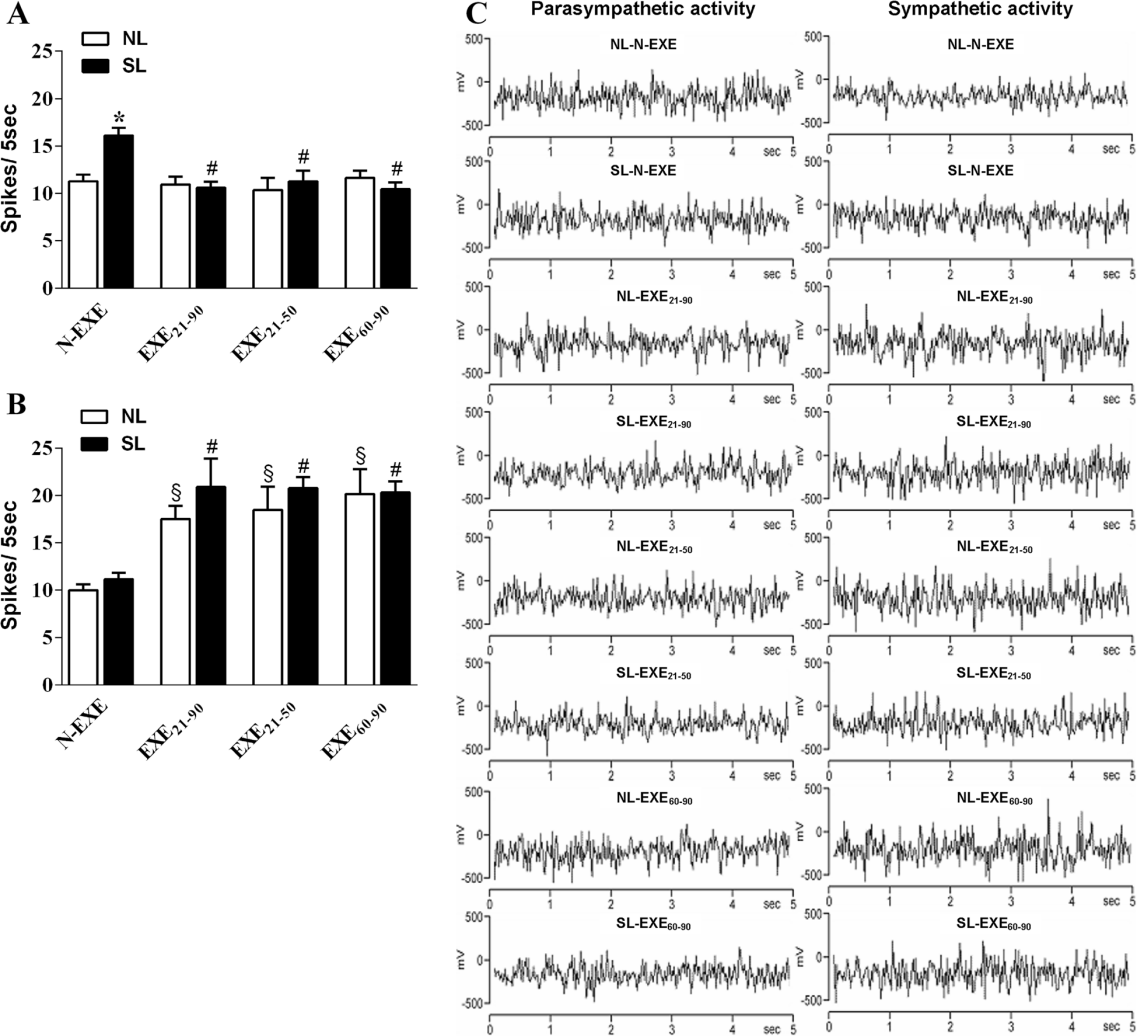
**Electrical activity of the autonomic nervous system.** All values are expressed as the mean ± SEM of 10–18 rats of each experimental group. The vagus **(A)** and greater splanchnic nerve **(B)** electrical activity. Symbols on the lines as well as letters on the bars represents the statistical difference by one-way ANOVA followed by Tukey’s test among groups. *p < .01 for SL-N-EXE v.s*.* NL-N-EXE; ^#^p < .01 for each one of SL-EXE group v.s*.* SL-N-EXE; ^§^p < .05 for each one of NL-EXE group v.s*.* NL-N-EXE. Representative records of each nerve discharge, which illustrate the data for each experimental group, are given in the Figure 3**C**.

The SL-N-EXE group exhibited a 31% increase in the vagus nerve firing rate when compared to the NL-N-EXE group (p < .05; Figure 3A). While the low-intensity and moderate exercise training did not cause any significant modifications in the number of vagus nerve spikes in the NL rats (NL-EXE_21–90_, NL-EXE_21–50_ and NL-EXE_60–90_ groups); a significant decrease in vagus nerve electrical activity was observed in the SL rats (SL-EXE_21–90_, SL-EXE_21–50_ and SL-EXE_60–90_ groups) when compared to their respective no-exercised groups (p < .01; Figure 3A).The sympathetic activity is showed in the Figure 3B, demonstrating that low-intensity and moderate exercise training increases the triggering rate of the greater splanchnic nerve by two-fold in both the NL and SL rats compared to their respective no-exercised groups (p < .05). No change was observed in the number of greater splanchnic nerve spikes in the SL-N-EXE rats when compared to the NL-N-EXE rats (Figure 3B). The representative records of each nerve discharge, which illustrate the data for each experimental group, are given in the Figure 3C.

## Discussion

As expected, a reduction in litter size during the suckling phase induced obesity in adult rats, as indicated by increased bw and increased fat tissue accumulation. Confirming data reporting that this experimental model of obesity is caused by the overfeeding behavior of young rats during lactation [30], this metabolic imprinting model displays glucose intolerance, insulin resistance, hyperphagia among others important metabolic disturbances [6,31].

The afferent vagus projects from the periphery to the nucleus of the solitary tract in the brainstem, a brain region situated in the dorsal vagal complex that functions as a port of entry for visceral information to the brain. Interestingly, the incoming peripheral signals about glucose levels can be modified by central glucose-sensing neurons at nearly every level of the central nervous system [32], and populations of neurons in the ventromedial and lateral hypothalamus are reported to increase their firing rates in response to the application of glucose [33].

The balance of the ANS is important to maintain constant glycemia. Overall, the parasympathetic stimulates insulin secretion, whereas the sympathetic inhibits it, which can produces decreases and increases in glycemia that are dependent on the glucose demand of cells, skeletal muscles and fat tissue. The data of the current research reveal, for the first time, that higher vagal nerve activity is observed in obese rats induced by early overfeeding. Our group also observed this feature in other different model of obesity [19,20,27,34], in all of these obesity model high fasting insulinemia and insulin resistance were observed. The method used in the present work cannot discriminate afferent from efferent signals; however, the firing rates from control rats are very similar to those reported by other authors [35,36]. Increased activation of the parasympathetic branch and/or reduced outflow of the sympathetic branch have been suggested to be responsible, at least in part, for the insulin oversecretion. Thus, in the current work we suggest that autonomic dysfunction could be indirectly responsible by the large fat pad accumulation in the SL rats, through the insulin lipogenesis action.

The most important find in the present work, is the observation that ANS may be modulated by the low-intensity and moderated exercise training, even in rats ran until puberty, and rats that start to run at begin of adulthood that includes later stages of developmental plasticity. Interestingly, using the swimming training protocol at the same periods of life that were used in the present work, we showed that MSG-obese mice displayed the metabolic ameliorations, however it was more prominent in mice that began to swim at weaning and stopped to do it at the end of puberty or at 90-day-old. Swimming training protocol did not improve the metabolic changes in mice swam between 60- and 90-day-old, like as is observed in early stages of life [24]. In agreement with, it has been demonstrated that exercise applied immediately after weaning is able to improve the cognitive ability of rats and it is correlated with high neuronal density in the neurons of the hippocampal area [37,38]. Concerning, in previous studies we reported that the puberty is one important phase of life in which metabolic changes can happen similar to those occur early in perinatal phases [19,20], which can be an important window to either malprogramming or deprogramming the metabolism.

It is known that physical exercise is a potent attenuator of obesity, activating energy expenditure, promoting lipolysis and increasing the consumption of fatty acids by peripheral tissues to reduce body fat deposits [39-41]. The peripheral metabolic adaptations promoted by physical exercise are activated by the hypothalamic neural pathways involved in the regulation of the sympathetic nervous system [40]. Our data demonstrate that physical exercise was able to improve the imbalanced parasympathetic activity of SL-obese rats, which was observed to be closely associated with reduction on the fat pad deposition in these obese rats. Interestingly, beyond high vagus nerve tonus no difference was observed in sympathetic activity of these overfeeding rats. On the same line the improvement of vagus nerve tonus was able in ameliorate the disrupted glucose homeostasis and fat pad stores, independent of the time exercise training protocol had begun.

In previous studies, using the same exercise training protocol applied throughout life, we showed that obese rats induced by high-fat diet restores the imbalanced autonomic function beyond other metabolic dysfunctions [27]. Similar results were obtained in rats fed hypercaloric diets that ran voluntarily [39].

Although our study to be a phenomenological study, our data are suggestive that autonomic changes are modulating the increased energy expenditure, the mobilization of fat stores, and the reduction in bw. The current work demonstrates that low-intensity and moderate exercise training is able to improve the glycemia, either in early- or late-exercised rats similar to NL rats. Even SL rats whose exercise training was stopped at the end of puberty, and SL rats that began to be trained at begin of adulthood, exhibited improvement of all metabolic impairment observed in the no-exercised SL-obese rats. These metabolic changes are acquired due to early training, especially during perinatal and puberty, because the brain is still forming, which could be also happen at begin of adulthood. Therefore, any stimulation of the abnormal nervous system activity, especially the ANS, contributes to a body spender phenotype.

In fact, to making a parallel with human condition, a body of data in the present work could suggest that a continual moderate walks and/or slow running, since moderate and low-intensity aerobic training, might help obese young children to reach a well health condition by preventing fat pad stores accumulation, heart diseases and/or type 2 diabetes. However, it is need to have caution regarding to make some paradigms between the exercise training in rats and in human. On this line, the necessity to have more experimental and epidemiological data, to do more precise recommendation about that exercise training to children is very important.

## Conclusion

These results demonstrate that low-intensity and moderate exercise training, independent of period that begin or stop improves the vagus nerves activity in adult-obese rats early programmed by overfeeding during suckling phase; and this exercise protocol provokes increased activity of the greater splanchnic nerve in both lean and SL-obese rats. Thus, the body of data in the current study highlights that low-intensity and moderate exercise training, independent of the age it could to be applied, can be one important no pharmacological tool against the metabolic syndrome problems that threat the human health around the word, specially childhood obesity, once it is a great risk factor to adulthood metabolic syndrome.

Regarding this point, more clinical and/or experimental studies should be performed to better explain the molecular pathways involved on interaction of exercise training on the ANS action. Given that, it could be one essential pharmacological target greatly important to improve health problem around the world.

## Abbreviations

AUC: Area under the curve; ANS: Autonomic nervous system; bw: Body weight; ivGTT: Intravenous glucose tolerance test; MSG: Monosodium L-glutamate; NL: Normal litter; NL-N-EXE: Normal litter no-exercised; NL-EXE: Normal litter exercised; NL-EXE_21–90_: Normal litter exercised from 21- to 90-days-old; NL-EXE_21–90_: Normal litter exercised from 21- to 50-days-old; NL-EXE_60–90_: Normal litter exercised from 21- to 90-days-old; SL: Small litter; SL-N-EXE: Small litter no-exercised; SL-EXE_21–90_: Small litter exercised from 21- to 90-days-old; SL-EXE_21–90_: Normal litter exercised from 21- to 50-days-old; SL-EXE_60–90_: Normal litter exercised from 21- to 90-days-old; VO_2max_.: Maximal voluve of oxygen uptake.

## Competing interests

The authors declare that they have no competing interests.

## Author’s contributions

WR, RMG, DXS, ABT and GSF designed the study and acquired the data; SG, AP, LFB and TAR interpreted and analysed the data; PCFM and JCde O drafted and wrote the manuscript; JCde O, TAR, LFB and PCFM revised intellectual and critically the manuscript. All of the authors approve the final version of the manuscript.
